# Mucosa-Associated Lymphoid Tissue Lymphoma of the Larynx

**DOI:** 10.1155/2020/8703921

**Published:** 2020-08-18

**Authors:** Omar Rizvi, Tyson Nielsen, Shethal Bearelly

**Affiliations:** Department of Otolaryngology, University of Arizona, Tucson, AZ, USA

## Abstract

**Background:**

Mucosa-associated lymphoid tissue (MALT) lymphomas are a subtype of non-Hodgkin lymphoma stemming from marginal zone B-cells. In this case report, we present two patients with an extremely rare localization of MALT lymphoma to the larynx.

**Methods:**

Case 1 is of a 78-year-old male presenting with a six-month history of progressive hoarseness with a past medical history significant for marginal zone lymphoma of the right orbit. Diagnosis was confirmed with a biopsy for extranodal marginal zone B-cell lymphoma of MALT type. An FDG-PET scan was done but did not show any sign of FDG avid malignancy, including at the primary site. Case 2 is a 60-year-old female presenting with one year of worsening throat discomfort, intermittent cough, and dyspnea with exertion. Pathology confirmed a diagnosis of extranodal marginal zone B-cell lymphoma of MALT type.

**Results:**

Case 1 was treated with low-dose radiation at 4 Gy delivered over two fractions of 2 Gy each. Upon completion of radiation treatment, he reported a resolution of his hoarseness and normalization of his voice. A four-month follow-up in May 2018 with flexible nasolaryngoscopy revealed a normal exam with fully mobile vocal folds bilaterally and no evidence of left false vocal fold submucosal mass. At seven months following treatment, the patient died unexpectedly of unknown causes. Case 2 was treated with radiation at 30 Gy in 15 fractions over the course of one month. Following completion of radiation therapy, she had improvement of her sore throat, nausea, dysphagia, dysgeusia, and dry mouth. At 21-month follow-up, she had no evidence of disease.

**Conclusion:**

This case report demonstrates that MALT lymphoma can present with much more benign and subtle symptoms. This highlights the importance of clinicians to keep broad differentials and consider MALT lymphomas in the setting of laryngeal masses.

## 1. Introduction

Mucosa-associated lymphoid tissue (MALT) lymphomas are a subtype of non-Hodgkin lymphoma stemming from marginal zone B-cells [1]. MALT lymphomas are low-grade lymphomas that generally remain localized with low potential for metastasis. However, recurrence and relapse of MALT lymphoma may be higher than documented due to long relapse periods and limited available data on this topic [2, 3]. It is mainly present in the stomach but can occur in a variety of locations such as the thyroid, breast, salivary glands, and orbit [4]. One of the rarest reported localizations of MALT lymphoma is in the larynx with approximately 30 cases reported since first reported by Diebold et al. in 1990 [4, 5].

Treatment for the most common MALT lymphoma subsite, gastric, is well documented and predicated on its association with and eradication of *H. pylori.* Less common subsites for MALT lymphomas have been treated with localized radiation, systemic chemotherapeutic agents, and depending on the location and surgery [2, 6, 7]. Since laryngeal MALT lymphoma is rare, the treatment approach is not well established. The treatment paradigm has largely been extrapolated from other subsites, but there is insufficient evidence to guide practitioners. In this case series, we describe the presentation and treatment of two patients with primary laryngeal MALT lymphoma and review the current literature for extranodal marginal zone lymphoma as it pertains to the head and neck.

## 2. Case 1

A 78-year-old man presented with a six-month history of progressive hoarseness denying dysphagia, odynophagia, hemoptysis, otalgia, or dyspnea. He was initially treated for a marginal zone lymphoma of the right orbit 2 years prior. Given his concern for vision loss, a low-dose radiation strategy of 4 Gy over 2 fractions was used, and he had a complete clinical response. He had been doing well until his new onset of hoarseness.

**Figure 1:**
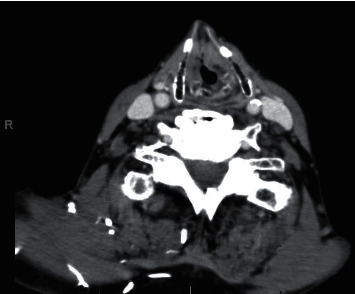
Axial CT neck with contrast demonstrating fullness of the left glottis.

A CT with contrast was obtained showing a tumor involving the left larynx at the level of the anterior false vocal fold extending into the laryngeal ventricle, true cords, and with a mild subglottic component (Figure 1).

**Figure 2:**
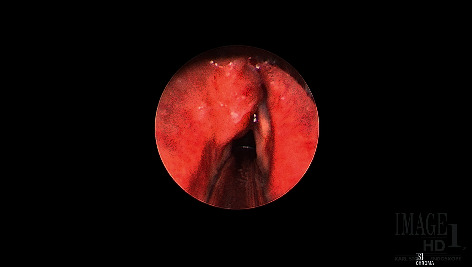
Rigid endoscopic view of the left false vocal cord mass.

Further evaluation was conducted with in-office flexible nasolaryngoscopy that confirmed a submucosal mass visible on the left false vocal cord. He then underwent microdirect laryngoscopy and biopsy of the mass (Figure 2).

Analysis revealed small monocytoid B-cells positive for CD20 with aberrant CD43 expression and negative for CD3, CD5, CD10, and BCL-6, confirming a diagnosis of extranodal marginal zone B-cell lymphoma of MALT type.

A staging FDG-PET scan did not show any sign of FDG avid malignancy, including at the primary site. Following multidisciplinary tumor board review, since he responded well to low-dose radiation to his orbit, he was again recommended to undergo low-dose radiation 4 Gy/2fx to his larynx. After completion of radiation treatment, he reported a resolution of his hoarseness and normalization of his voice. At four-month follow-up, repeat in-office flexible nasolaryngoscopy revealed a normal exam with fully mobile vocal folds bilaterally and no evidence of recurrence or persistence. He also had a PET-CT scan which confirmed he had no evidence of disease. Unfortunately, at 7 months following treatment, the patient died unexpectedly of unknown causes.

## 3. Case 2

A 60-year-old woman presented with one year of worsening throat discomfort, intermittent cough, and dyspnea with exertion. She denied any hemoptysis, otalgia, or odynophagia. She had no other significant past medical or social history including no history of tobacco use.

**Figure 3:**
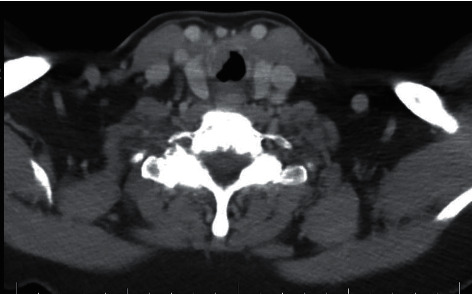
Axial CT neck with contrast demonstrating a right subglottic mass.

A CT neck with contrast demonstrated a mass involving the anterolateral right subglottic larynx with extension along the upper cervical trachea (Figure 3).

**Figure 4:**
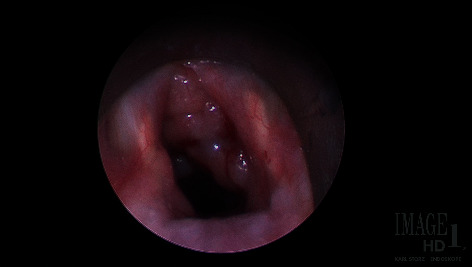
Rigid endoscopic view of the right subglottic mass.

In-office flexible nasolaryngoscopy identified a fleshy pink-colored mass in the immediate right subglottis obstructing approximately 50% of the airway. She then underwent microdirect laryngoscopy and bronchoscopy for debulking and biopsy (Figure 4).

Operative findings were notable for extension of the mass approximately 1 cm below the true vocal fold.

Pathologic analysis noted small B-cells positive for CD19, CD20, and PAX5 and negative for CD5, CD10, and cyclin D21. FISH was negative for the 18q21 MALT1 gene. The combination of these findings confirmed the diagnosis of extranodal marginal zone B-cell lymphoma of MALT type was made.

A staging CT neck/chest/abdomen/pelvis scan was negative for metastatic disease. Following multidisciplinary tumor board review, she was recommended low-dose radiation at 24–30 Gy in 2 Gy daily fractions for approximately 3 weeks contingent upon review of a bone marrow biopsy. Bone marrow biopsy was negative, and radiation treatment was finalized as 30 Gy in 15 fractions over the course of 1 month. Treatment was completed by May 2018. Following radiation treatment, she reported mild dysphagia and dysgeusia which has improved. Her breathing has improved, and she has returned to her normal exercise tolerance. At 21-month follow-up, there was no evidence of disease on exam or imaging; however, she continues to report a mild cough intermittently with no issues with eating, drinking, or breathing.

## 4. Discussion

Although we would expect patients with laryngeal MALT lymphomas present with severe dysphagia and dysphonia, these cases demonstrate that they can present with just subtle symptoms.

Squamous cell carcinoma is the most common malignancy within the larynx, but clinicians need to keep a broad differential and consider MALT lymphomas. There are little data about how to treat laryngeal MALT lymphoma, and reporting these two cases helps clinicians identify and manage this rare disease.

Following initial presentation, per our institutional preference, a CT neck scan with contrast was done to assess extent of the primary malignancy. In the first patient, staging was done with a FDG-PET scan. Though no metastasis was identified, the primary malignancy was also not visualized on the scan. There is debate about the sensitivity of FDG-PET scans in diagnosing and staging MALT lymphomas, with some studies recommending it, while others find it lacks sensitivity [4, 8, 9]. The uncertainty over its utility forces clinicians to consider bone marrow biopsies for complete staging.

Immunophenotype can be determined either by immunohistochemistry or by flow cytometry, and MALT lymphomas are usually positive for CD19/20/22/79 and negative for CD5/10/23/BCL-6 and cyclin D1 [1]. Both patients demonstrated CD20 positivity and CD5 negativity, confirming the diagnosis.

Immunohistochemistry was in line with the current literature as both were at least CD20 positive and CD5 negative, which is a characteristic of most MALT lymphomas.

In the second patient, FISH studies were negative for the 18q21 MALT1 gene, which has been associated with *H. pylori*-independent gastric MALT lymphoma [10]. This association with *H. pylori*-independent gastric MALT lymphomas might point to an inherent genetic cause of MALT lymphomas, gastric or otherwise [10]. Additionally, this translocation has other implications as well such as a poor response to antibiotics, advanced disease, and a lower risk of transforming to diffuse large B-cell lymphoma [11].

Since laryngeal MALT lymphoma is rare, its pathophysiology has been extrapolated from our knowledge of other subsites. The most common type of MALT lymphoma is gastric with one recent study by Khalil et al. in 2014 [12] reporting an incidence rate double that of the second most common MALT lymphoma, spleen [11]. As such, gastric MALT lymphoma provides a better documented basis from which to understand the connection between MALT lymphoma and chronic inflammation. While the association between gastric MALT lymphoma and *H. pylori* is well established, the underlying pathophysiology has only recently started to be understood. A recent review by Saegert et al. proposes a three-step process by which chronic inflammation induced by *H. pylori* infection may lead to MALT lymphoma. The first step arises from *H. pylori* infection leading to lymphoid infiltration and antigen stimulation through somatic hypermutation of immunoglobulin heavy chains [13]. The second step consists of genetic mutations at specific locations ranging from trisomy of chromosomes to various translocations [12]. The final step involves the dysregulation of the transcription factor NF-*κ*B potentially allowing for unchecked B-cell proliferation and lymphoma pathogenesis [12]. With MALT lymphoma of the larynx, the pathophysiological association with *H. pylori* is not established. The rarity of this presentation of MALT lymphoma makes producing any kind of association with *H. pylori* not possible currently [4].

MALT lymphomas also have a strong association with certain autoimmune disorders such as Sjögren's syndrome, Hashimoto's thyroiditis, and systemic lupus erythematosus [13]. Sjogren's syndrome shows an association with MALT lymphoma in the parotid gland with some studies finding a 30-fold increased risk of MALT lymphoma [14]. Hashimoto's thyroiditis is associated with MALT lymphoma in the thyroid gland [13, 14]. Systemic lupus erythematosus does not show a preference for specific location but does have an overall increased risk of MALT lymphoma [14]. The relationship between these autoimmune conditions and MALT lymphoma reflects the direction of recent research suggesting that the pathophysiology may be induced by chronic inflammation predisposing to progression to lymphoma [14].

The variability in risk and location in these lymphomas is hypothesized to have a “dose-response” relationship, with each autoimmune condition producing varying amounts of inflammation and accounting for different risks of developing MALT lymphoma [14].

A better understanding of the pathophysiology also underlies the difference in treatment between gastric MALT lymphoma and laryngeal MALT lymphoma. For localized *H. pylori*-positive gastric MALT lymphoma, treatment is to eradicate *H. pylori* which can be done using any of the established antibiotic regimens such as “triple therapy” for 7–14 days with proton pump inhibitors (PPIs), amoxicillin, and clarithromycin [15]. If *H. pylori* persists despite medical therapy, irradiation and systemic therapies can be used [15]. Radiation therapy typically has good outcomes but is only used in severe and resistant *H. pylori* infections [15]. In comparison, most cases of MALT lymphoma of the larynx are not known to have *H. pylori* infection and are treated initially with radiation instead of antibiotics, though the optimal dose of radiation is unknown [6]. Treatment for severe or recurrent cases is still controversial with approaches varying from observation to systemic intervention with drugs such as chlorambucil or rituximab [6].

In summary, we present two cases of laryngeal mucosa-associated lymphoid tissue lymphoma, detail the presentation and treatment, and review the most up-to-date literature regarding this disease. Both cases had just subtle signs and symptoms of disease, so clinicians need to keep a broad differential to correctly identify the pathology. The pathophysiology is unknown but perhaps is related to chronic inflammation or autoimmunity. Diagnosis is made with a biopsy and confirmatory immunohistochemical testing for CD20 and CD5. Treatment for laryngeal MALT lymphoma is evolving, but the mainstay appears to be low-dose radiation therapy. Long-term outcomes and prognosis are unknown because of the rarity of this disease, and further reporting and research regarding this disease are needed.

## Data Availability

The data for both these cases were accessed through the electronic medical record. They are not available for readers to review as they contain confidential patient health information.
